# Case of Cancer of the Uterus

**Published:** 1831-04-01

**Authors:** 


### Case of Cancer of the Uterus. By John Beatty, M.D. &c.*

Rachael Collins, ætatis 54, a domestic, complained in May, 1828, that for some years past she had suffered from profuse uterine discharges of blood at irregular periods, attended with some pain round her loins and down her thighs, with loss of appetite and debility. When the flow of blood was absent, a most offensive and scalding discharge of greenish yellow matter took place, excoriating the labia and adjoining parts. On examination the os uteri was found low in the vagina in a perfectly carcinomatous state, with a very large cauliflower-like surface, painful to the touch. She had borne two children when young, but for several years past had not cohabited with her husband. Dr. Beatty prescribed for the patient, and saw nothing more of her till July, 1829. All the symptoms were now very much increased, but her chief suffering, was from inability to pass her stools from what she termed a lump in her passage, and almost incessant vomiting. Mild injections relieved her for a time, but soon the increase of the tumour rendered it impossible to throw up any fluid. The next distressing symptom that presented itself was the passage of the stools from the vagina, and the patient lived in a miserable state till the 18th of September, 1829.

*Sectio Cadaveris*. Body much emaciated. On opening the abdomen the large intestine was found contracted, and nearly empty. A convolution of the ileum, near its termination, dipped down into the pelvis, and passed in a curved line from left to right, between the upper and back part of the vagina and the rectum, to both of which it adhered. The urinary bladder was dilated, but empty, and the peritoneum covering its upper and back part was very thick and strong. The contents of the pelvis were detached from its walls, but much difficulty was experienced on the left side, in consequence of the rectum and vagina being connected to it by a firm mass which it was necessary to cut through. The uterus appeared very little enlarged. but felt firm, and a number of minute blood-vessels were seen ramifying on its surface. The vulva was dilated, and the mucous membrane lining the vagina and labia was of a dark chocolate colour, and thickened. A fetid purulent discharge oozed from the vagina. On slitting up the back of the rectum, the lining membrane presented the same dark appearance as the inte-

---

* Dublin Medical Transactions, Vol. 1, Part 1.

rior of the vagina. The anterior surface was perforated by an irregular ulcerated opening, about an inch in diameter, the edges of which were thick and hard, and the adjoining part of the bowel partook of the same carcinomatous character. This opening communicated directly with the vagina, to which the rectum was here firmly attached; in fact these two, with the portion of the ileum described, were all blended into one mass of cancerous tumour, which, on opening the vagina, was found to proceed from the os uteri. The ileum, like the rectum, had a free communication with the vagina at the point of adhesion. The back of the bladder had also been implicated, and a large opening between it and the anterior part of the vagina was discovered, the edges of which were thickened and irregular. The mucous membrane of the bladder was thickened, and that of the urethra had the dark appearance that was found in the rectum and vagina. The body of the uterus when cut into presented a dense white cartilaginous surface; its cavity was filled with pus, and the lining membrane very vascular. From the os uteri a large cancerous mass projected, which adhered to all the parts in its vicinity, and had produced the ulcerated communications described.

After the paper on cancer uteri by Dr. Montgomery, an analysis of which we have given in our last Number, we need say little on the present case. But Dr. Beatty makes one remark which we cannot pass in silence.

"This case is in perfect accordance with an observation I have made for a great number of years, that in almost every instance where I have been consulted for cancer of the uterus, the disease has arisen in persons who, while young, had either lost their husbands, or separated from them. I do not remember to have met with an instance of the disease, in which an early interruption of connubial intercourse had not taken place. A remarkable case occurred to me in 1814, in which I acted upon this principle, and by recommending a restoration of conjugal rights, succeeded in checking the disease.

A lady and her husband, after having had children, had lived very much asunder for some years, and at the time I have mentioned, I was consulted by the lady, in whom incipient cancer was now evident. She complained of pain and weakness in the loins, so great as almost to incapacitate her from walking; this was accompanied with a sense of bearing down, and a leucorrhœal discharge.—Acute pain shot from time to time across the pelvis, and the digestive organs were very much deranged. The os uteri was found lower in the vagina than is natural, and presented a thickened, irregular, and indurated surface, painful to the touch. The upper part of the vagina was also hard to the feel, and the rugæ were considerably obliterated. A consultation was held with two physicians of the most extensive experience in this kingdom, to whom I reported the result of my examination. One of the gentlemen having made a similar examination, confirmed my report and opinion, and they both agreed in recommending a total separation of beds, as the plan most likely to prolong a life which must become a sacrifice.

I mentioned the observations I had made on patients labouring under cancer of the uterus, and expressed a hope that if connubial intercourse were restored, the progress of the disorder might be arrested. The idea was new to them, but they readily acceded to my proposal. The husband returned to his wife's bed, and the result was, the birth of a healthy child in less than a year.

A perfect restoration to health followed, which has continued without interruption, though fourteen years have elapsed since the child was born. The lady, from having been emaciated and worn down, recovered her flesh and good looks, and has mixed freely in the upper class of society ever since."

